# Changes in Salivary Oxytocin Level of Term Pregnant Women after Aromatherapy Footbath for Spontaneous Labor Onset: A Non-Randomized Experimental Study

**DOI:** 10.3390/ijerph20136262

**Published:** 2023-06-30

**Authors:** Yuriko Tadokoro, Kaori Takahata, Takuya Shuo, Kazuyuki Shinohara, Shigeko Horiuchi

**Affiliations:** 1 Chiba Faculty of Nursing, Tokyo Healthcare University, Funabashi 273-8710, Japan; 2 School of Nursing, Shonan Kamakura University of Medical Sciences, Kamakura 247-0066, Japan; k.takahata@sku.ac.jp; 3 Faculty of Health and Medical Sciences, Hokuriku University, Kanazawa 920-1180, Japan; t-shuo@hokuriku-u.ac.jp; 4 Graduate School of Biomedical Sciences, Nagasaki University, Nagasaki 852-8523, Japan; kazuyuki@nagasaki-u.ac.jp; 5 Women’s Health and Midwifery, School of Nursing Science, St. Luke’s International University, Tokyo 104-0044, Japan; shigeko-horiuchi@slcn.ac.jp

**Keywords:** aromatherapy, salivary oxytocin, salivary cortisol, pregnant women, complementary and alternative medicine, clary sage essential oil, lavender essential oil, jasmine oil, footbath, stimulation of labor

## Abstract

Background: Aromatherapy is usually used to stimulate labor. However, its specific physiological effects have been scarcely examined. We evaluated whether an aromatherapy footbath increases oxytocin levels in term pregnant women. Methods: In this quasi-experimental study, low-risk term pregnant women in Japan underwent aromatherapy using a footbath (1) infused with clary sage and lavender essential oils, (2) infused with jasmine oil, or (3) with no infused oils (control group). The primary outcome was the salivary oxytocin level. The secondary outcomes were uterine contractions and cortisol levels. Results: In the clary sage and lavender group (*n* = 28), the oxytocin level increased significantly after the footbath (*p* = 0.035). The jasmine group (*n* = 27) and control group (*n* = 27) exhibited trends toward a respective increase and decrease in the oxytocin level; however, the changes in the oxytocin levels between the clary sage and lavender group and the control group showed no significance difference. There were no significant differences in the changes in the uterine contractions and cortisol levels between the experiment and control groups. Conclusions: The changes in the oxytocin levels in the clary sage and lavender group did not differ significantly with those in the control group, possibly because of the small sample size. Further studies are required to examine the effects of repeated aromatherapy footbaths to stimulate labor.

## 1. Introduction

Normal physiologic childbirth is characterized by the spontaneous onset and progression of labor. The benefits to women and their babies include effective labor pattern, successful lactation, and improved bonding behavior between the mother and her baby. The spontaneous onset of labor is a key factor for achieving normal physiologic childbirth, which can be disrupted by medical induction of labor [1]. Nevertheless, medical induction of labor is often performed in 25–29% of women in developed countries to prevent post-term delivery [2,3]. Notably, pregnant women prefer receiving information on complementary and alternative medicine (CAM) for stimulating labor before undergoing medical induction of labor [4]. Aromatherapy is one of the CAMs used to stimulate labor. It is usually applied in a footbath, via scent inhalation, or by massage using clary sage, lavender, or jasmine oil [5,6,7,8,9,10]. 

In medical induction of labor, synthetic oxytocin is infused to induce uterine contractions and the subsequent onset and augmentation of labor [11,12]. Similarly, aromatherapy for stimulating labor is considered to be associated with the release of hormonal oxytocin. Our recent feasibility study showed that the inhalation of clary sage oil tended to increase the oxytocin level of term pregnant women [13]. In postmenopausal women, oxytocin levels were shown to increase after the inhalation of clary sage, lavender, or jasmine oil [14]. Lavender or jasmine oil has also been reported to shorten the duration of labor and delivery [9,15,16,17]. Shortening the duration of labor and delivery requires uterine contractions which are induced by oxytocin. Therefore, aromatherapy can be considered to increase the release of oxytocin. In Japan, footbaths to stimulate labor are used in combination with clary sage and lavender oils in some clinical settings. Lavender oil is suggested to lessen stress [18], which is very important as stress during breastfeeding suppresses oxytocin release [19]. Therefore, the combination of clary sage and lavender oils may increase the oxytocin level by inhibiting the stress-induced suppression of oxytocin release.

A systematic review on the adverse events of aromatherapy [20] has shown no adverse events with the single use of clary sage or jasmine oil. Although eczema or dermatitis after the topical use of lavender oil has been reported, the skin sensitization is categorized as moderate and there are no contraindications with its use [21]. The combination usage of clary sage and lavender oils in footbath has shown no adverse events [22,23,24]. The risk of skin sensitization using these oils in a footbath is considered to be very minimal because of the low concentration of exposure on the skin. 

The effects of aromatherapy in reducing labor pain and anxiety in term pregnant women have also been investigated [9,15,16,17,25,26,27,28]. However, the effects of aromatherapy in stimulating the onset of spontaneous labor remain scarcely investigated, as there have been limited reports and reviews on such effects to date [7,8,10,22,23,24,29,30,31]. In the present study, we clarified the effects of an aromatherapy footbath prepared for enhancing the spontaneous onset of labor on the changes in the salivary oxytocin level of term pregnant women. We specifically elucidated whether an aromatherapy footbath infused with clary sage and lavender oils and an aromatherapy footbath infused with jasmine oil will increase the salivary oxytocin level of term pregnant women after using the footbaths compared with a footbath with no infused essential oils.

## 2. Materials and Methods

### 2.1. Study Design

We conducted a non-randomized experimental study using two experimental groups, namely, a clary sage and lavender oil group (aromatherapy footbath with clary sage and lavender oils), a jasmine oil group (aromatherapy footbath with jasmine oil), and a control group, namely, the no essential oil group (footbath with no essential oils). This study was approved by the Research Ethics Committee of St. Luke’s International University (No. 15-084) and was carried out in accordance with the ethical standards set forth in the Helsinki Declaration of 1975. Written informed consent was obtained from all the participants. This study was registered in the Clinical Trials Registry of University Hospital Medical Information Network in Japan (https://center6.umin.ac.jp/cgi-open-bin/ctr_e/ctr_view.cgi?recptno=R000023980, accessed on 28 June 2023).

### 2.2. Participants and Setting

Participant recruitment and footbath intervention were conducted at a single birth center in Tokyo, Japan, between February 2016 and July 2016. The inclusion criteria were women who were (a) at low risk and had a singleton cephalic presentation pregnancy between 38 and 40 gestation weeks before labor onset, (b) planning vaginal birth, (c) between the ages of 20 and 40 years, and (d) able to communicate, read, and write in Japanese. All women who met the inclusion criteria were recruited. 

The exclusion criteria were women with the following background: (a) a history of obstetric complications (e.g., post-term delivery, cesarean section, recurrent miscarriage and pregnancy loss, and abnormalities during pregnancy) and a medical history (e.g., dermatitis and endocrine, olfaction, and mental disorders), (b) planning labor induction, (c) with alcohol addiction, (d) smokers and those having allergy to foods, medicine, plants, and essential oils, and (f) currently breastfeeding. 

### 2.3. Masking

Participants were masked as to whether the footbath was infused with clary sage and lavender oils, infused with jasmine oil, or with no infused oils. However, they could make an assumption whether they were in the experiment or control group by the scent from the start of the footbath intervention because of the nature of the experiment. Healthcare providers at the study settings and a biologist who assayed the laboratory outcomes were masked regarding the participant allocations. The participant allocations were not shown to the healthcare providers and the medical records were not written. Saliva samples were controlled only using an orderly numbered ID. 

### 2.4. Sample Size

The sample size was calculated based on our previous study measuring the changes in salivary oxytocin level after aromatherapy in pregnant women [13]. By calculation with 7.0 pg/mL of the detected change in oxytocin level and 7.0 pg/mL of its deviation, 15 subjects per group were estimated with a two-sided 5% significance. Given that the estimated percentage of missing samples caused by insufficient saliva volume for measuring oxytocin level was 27%, 20 subjects were required per group. Because of the limitations of the study period and the number of participants, the participants used two of the three footbaths on separate days, and the data analysis was based on the footbath groups. Therefore, 30 participants were estimated to be required for the entire footbath groups. Beyond the estimation of missing samples, some participants could not drool a sufficient volume of saliva for oxytocin level measurement and gave birth before the intervention. Consequently, some participants were added, and the recruitment was closed when a sufficient number of samples was obtained. 

### 2.5. Interventions

#### 2.5.1. Footbath Preparations in Experimental and Control Groups

For the footbath preparations, 10.7 L of water was placed in a warm sustainable footbath device. The water depth was 15 cm, and the footbath temperature was set to 42 °C. 

In the experimental groups, immediately before using the footbaths, essential oils were first mixed with 6 g of salt to emulsify the oil in water, and then the essential oils were infused into the footbath water. In the clary sage and lavender group, 0.2 mL of clary sage essential oil (Lot No. 57, Tree of life Co., Ltd., Tokyo, Japan) and 0.1 mL of lavender essential oil (Lot No. 73, Tree of life Co., Ltd.) were used. In the jasmine group, 0.25 mL of jasmine absolute oil (Lot No. 39, Tree of life Co., Ltd.) was used. The dilutions of the essential oils in the footbaths were confirmed beforehand by five non-pregnant women as the strongest scent as well as a comfortable scent when using the footbaths for 20 min. 

Clary sage essential oil is extracted by steam distillation of the flowers and leaves of *Salvia sclarea* (Lamiaceae) and contains linalyl acetate (55.8%), linalool (23.5%), alpha-terpineol (3.3%), beta-caryophyllene (2.1%), geranyl acetate (2.1%), germacrene D (1.1%), and sclareol (0.6%) [32]. Lavender essential oil is extracted by steam distillation of the flowers and leaves of *Lavandula officinalis* (Lamiaceae) and contains linalyl acetate (37.9%), linalool (31.4%), cis-beta-ocimene (3.8%), terpinenenen-4-ol (2.7%), trans-beta-ocimenes (2.6%), acetic acid lavandulyl ester (2.0%), 3-octanone (1.1%), 1.8-cineole + p-mentha-1(7), 2-diene (1.0%), alpha-terpineol (0.9%), lavandulol (0.6%), and camphor (0.4%) [33]. Jasmine absolute oil is obtained by solvent extraction of *Jasminum officinales* (Oleaceae) and contains benzyl acetate (23.9%), benzyl benzoate (17.3%), linalool (6.7%), isophytol (5.3%), epoxy dihydrosqualene (2.8%), phytol (2.8%), methyl linolenate (2.6%), eugenol (2.3%), acetic acid (2E,7R,11R)-3,7,11,15-tetramethyl-2-hexadecenyl ester (2.1%), methyl hexadecanoate (1.8%), benzyl alcohol (1.74%), indole (1.63%), squalene (1.4%), cis-3-Hexenyl benzoate (1.3%), jasmone (1.3%), geranyl linalool (1.1%), and alpha-farnesene (0.8%) [34]. Aromatherapy oils are categorized as sundries and can be bought and used by anyone in Japan.

In the control group, the footbath was similarly prepared but was only infused with 6 g of salt.

#### 2.5.2. Procedures

The footbath intervention was performed separately for each participant in a quiet room (17.8 m^3^) between 12:00 and 14:00 on a weekday and lasted for 1.5 h. Air condition was controlled at 50% room humidity and 25 °C. The lead researcher (Y.T.) or a research assistant confirmed these settings and all the procedures following the intervention protocol.

The participants were asked to avoid any activities that could affect the assay of salivary oxytocin and cortisol levels or their baseline levels [35] until the intervention. At the time of the intervention, the participants initially prepared for saliva collection for the measurement of oxytocin and cortisol levels and then for the aromatherapy footbath. The preparations involved the following activities: rinsing of the mouth with tap water, checking of fetal heart rate for 30 s, receiving instructions on saliva collection, answering a self-administered questionnaire including their characteristics, drinking 100 mL of water, and watching a silent train movie to standardize the participants’ setting. 

The participants subsequently collected saliva 10 min after drinking water (pre-footbath saliva collection), used the assigned footbath for 20 min, and then collected saliva (post-footbath saliva collection). They drank 100 mL of water again 10 min before the post-footbath saliva collection. The footbath was continued until the post-footbath saliva collection was completed. 

Finally, the participants answered a self-administered questionnaire including questions regarding changes in their uterine contractions. The following were also checked: their fetal heart rate for 30 s and any skin symptoms in their feet which were immersed in the footbath. 

### 2.6. Outcome Measures

Some basic characteristics of women were collected using a self-administered questionnaire. These characteristics have been reported to affect the oxytocin level and include depression [36,37], anxiety [38], accuracy of gestation weeks (confirmation of the due date by ultrasonography before 12 weeks of gestation) [39], delivery history [40], marital status [41], educational level [42], body mass index ≥ 29 [43], age, and race [44]. Depression and anxiety were assessed using the Japanese version of the Center for Epidemiologic Studies Depression Scale (CES-D) [45] and State-Trait Anxiety Inventory (STAI) [46]. The CES-D consists of 20 items with a 4-point Likert scale in which a depressed condition is ascertained when the score is higher than 15. Cronbach’s α was shown as 0.8 in the CES-D. The STAI includes two scales, namely, trait (A-trait) and state (A-state) of anxiety. Each scale consists of 20 items with a 4-point Likert scale, and higher scores indicate higher anxiety. Cronbach’s αs were shown as 0.85 and 0.87 in the A-state and B-state, respectively.

#### 2.6.1. Primary Outcome: Changes in the Salivary Oxytocin Level before and after Using the Footbath

Salivary oxytocin level was measured using pre-footbath and post-footbath saliva samples. In each pre-footbath and post-footbath saliva collection, the participants initially accumulated saliva in their mouth for 3 min and then passively drooled the saliva in a prechilled polypropylene tube (Eppendorf, Hamburg, Germany) [47,48]. This procedure was conducted three times in each saliva collection to obtain at least 1.5 mL of saliva. The tubes containing the saliva samples were kept in ice during the saliva collection and then stored at −80 °C until the oxytocin enzyme-linked immunosorbent assay (ELISA; Enzo Life Sciences Inc., Farmingdale, NY, USA) in duplicates. 

#### 2.6.2. Secondary Outcome: Changes in the Subjective Uterine Contraction and Salivary Cortisol Level before and after Using the Footbath

The participants assessed the changes in their subjective uterine contractions from three selections in the self-administered questionnaire: increased, unchanged, and decreased. 

For the measurement of salivary cortisol level, the participants drooled at least 250 µL of saliva into a separate propylene tube after the collection of saliva for the measurement of oxytocin level. Salivary cortisol level was assayed by ELISA (Salimetrics, State College, PA, USA) in duplicates following the manufacturer’s product instruction. 

#### 2.6.3. Arrangement in Measurement of Salivary Oxytocin and Cortisol Levels

In the salivary oxytocin and cortisol level measurements, saliva samples from individual participants were arranged on the same ELISA batch. The inter-assay and intra-assay coefficients of variabilities (CVs) were reported as 2.6–13.3% and 11.8–20.9% for oxytocin [49] and 3–11% and 4–7% for cortisol, respectively [50]. Oxytocin and cortisol levels for intra-assay CVs that were higher than 10% were excluded from the analysis, based on the technical report of Salimetrics LCC. An analysis was conducted using data obtained from the pre-footbath and post-footbath interventions. 

#### 2.6.4. Adverse Events 

Any symptoms in the skin of the feet immersed in the footbath, abnormal fetal heart rate after the footbath intervention (<110 and >160 bpm), premature rapture of membrane (PROM), Apgar score < 7, and admission of the baby to the neonatal intensive care unit (NICU) were recorded during the intervention and identified using medical records.

### 2.7. Statistical Analysis 

Pre-footbath and post-footbath oxytocin and cortisol levels were graphically reviewed as normal distribution without outliers and then compared within each group using a paired t-test. Changes in the pre-footbath and post-footbath (mean difference: MD) salivary oxytocin and cortisol levels were compared between the experimental groups and the control group using an independent *t*-test. Welch’s test was used if Levene’s test for equality of variances was not confirmed. Changes in subjective uterine contractions were compared between the experimental groups and the control group using the chi-square test. As an additional analysis, the correlation between changes in salivary oxytocin and cortisol levels was also identified using Pearson’s correlation coefficient. The analysis was conducted per protocol without assigning missing values.

Data were expressed as mean and standard deviation (SD). A statistical analysis was conducted using SPSS version 24.0J for Windows with a two-sided 5% level of significance.

## 3. Results

**Figure 1:**
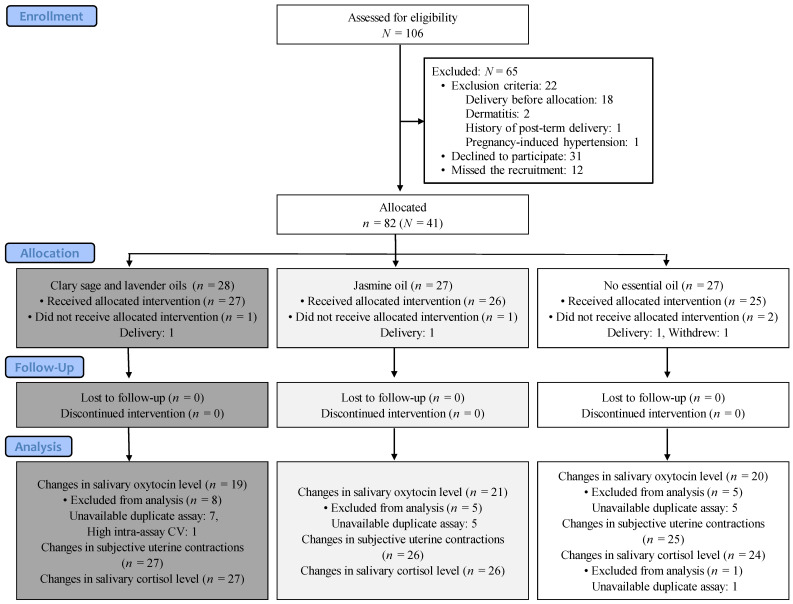
Flowchart showing intervention profile. N: number of participants; n: number of subjects allocated to each footbath group; CV: coefficients of variability.

Figure 1 shows a flowchart of the intervention profile. Of the 106 women who were assessed for eligibility of this study, 65 women were excluded; thus, a total of 41 women were included. Following the study method that one woman uses two footbaths of the three footbath groups, 82 participants were allocated. Three participants were excluded owing to changes in their conditions that did not meet the inclusion criteria, and one participant withdrew because of a personal circumstance. The final numbers of participants per group who received the interventions were as follows: 27 participants in the clary sage and lavender group, 26 participants in the jasmine group, and 25 participants in the no essential oil group. Each intervention was carried out as planned. Although no participant was lost to follow-up, there were some missing data owing to unavailable duplicate assays in the oxytocin level and cortisol level measurements and the high intra-assay CV in the oxytocin level. Therefore, the analysis was conducted in the clary sage and lavender oil group (*n* = 19), jasmine oil group (*n* = 21), and no essential oil group (*n* = 20) on the changes in the oxytocin levels; in the clary sage and lavender oil group (*n* = 27), jasmine oil group (*n* = 26), and no essential oil group (*n* = 25) on the changes in the uterine contractions; and in the clary sage and lavender oil group (*n* = 27), jasmine oil group (*n* = 26), and no essential oil group (*n* = 24) on the changes in the cortisol levels.

As shown in Table 1, the characteristics of the participants in the experimental and control groups showed no significant difference. 

### 3.1. Primary Outcome: Changes in Salivary Oxytocin Level before and after Using the Footbath

The pre-footbath salivary oxytocin level was not significantly different between the experimental and control groups (clary sage and lavender oil group vs. no essential oil group: *p* = 0.952; jasmine oil group vs. no essential oil group: *p* = 0.543). In the clary sage and lavender oil group, the salivary oxytocin level significantly increased from the pre-footbath level (139.5 pg/mL [106.5]) to the post-footbath level (152.0 pg/mL [115.2]) (MD 12.5 [23.9], *n* = 19, *p* = 0.035). This is also similarly increased in the jasmine oil group from 166.2 pg/mL [136.7] to 171.0 pg/mL [141.4] (MD 4.8 [51.8], *n* = 21, *p* = 0.676). In contrast, the salivary oxytocin level decreased in the no essential oil group from 141.6 (pg/mL) [118.3] to 138.4 (pg/mL) [91.4] (MD—3.3 [71.0], *n* = 20, *p* = 0.839) (Table 2). However, there was no significant difference in the changes in the salivary oxytocin levels between the experiment groups and the control group (clary sage and lavender oil group vs. no essential oil group: MD = −15.8, 95% CI for MD [−50.6, 18.9], t (37) = −0.92, *p* = 0.363; jasmine oil group vs. no essential oil group: MD = −8.1, 95% CI for MD [−47.2, 31.0], t (39) = −0.42, *p* = 0.679). Notably, the changes in the pre-footbath and post-footbath salivary oxytocin levels showed large standard deviations. 

### 3.2. Secondary Outcomes: Changes in Subjective Uterine Contraction and Salivary Cortisol Level before and after Using the Aromatherapy Footbath

Most of the women in all the groups had unchanged subjective uterine contractions. There was no significant difference in the changes in subjective uterine contractions between the experimental groups and the control group (Table 3). 

The pre-footbath salivary cortisol level was not significantly different between the experimental groups and the control group (clary sage and lavender oil group vs. no essential oil group: *p* = 0.870; jasmine oil group vs. no essential oil group: *p* = 0.748). The salivary cortisol level significantly decreased from the pre-footbath levels to the post-footbath levels in all the groups (clary sage and lavender oil group: *p* < 0.001; jasmine oil group: *p* = 0.001; no essential oil group: *p* < 0.001) (Table 4). There were no significant differences in the changes in the salivary cortisol levels between the experimental groups and the control group. 

The correlations between changes in the salivary oxytocin and cortisol levels were not significant in each group (clary sage and lavender oil group: r (17) = −0.138, *p* = 0.572; jasmine oil group: r (19) = 0.164, *p* = 0.476; no essential oil group: r (18) = 0.046, *p* = 0.553). 

### 3.3. Adverse Events 

There were no post-footbath skin symptoms in any of the groups. Of the 41 women, 10 women had PROM, which occurred after three days in all the interventions except in one woman. This woman underwent the intervention in the jasmine oil group and then in the control group. PROM occurred 61 h after the intervention in the jasmine oil group and 16 h after the intervention in the control group. There were no babies who had an abnormal fetal heart rate after the aromatherapy footbath, had an Apgar score <7, or were admitted to NICU.

## 4. Discussion

### 4.1. Primary Outcome: Changes in Salivary Oxytocin Level

The salivary oxytocin level significantly increased after using the footbath infused with clary sage and lavender oils. Moreover, the salivary oxytocin level showed an increasing trend after using the footbath infused with jasmine oil. To the best of our knowledge, the present study is the first to clarify changes in the salivary oxytocin level after using an aromatherapy footbath infused with essential oils in term pregnant women. These results are consistent with the results of a recent study on postmenopausal women [14]. That study showed a significant increase in the salivary oxytocin level after exposure to the scent of clary sage, lavender, or jasmine oil. 

Unfortunately, we are unable to definitively conclude that this increase in the salivary oxytocin level leads to spontaneous onset of labor. This is because the amount of increase in the salivary oxytocin level to stimulate the spontaneous onset of labor is unknown. Breast stimulation is one of the alternative methods that women use to stimulate the spontaneous onset of labor and is the only method shown to increase the spontaneous onset of labor [51] among other alternative methods, such as the use of acupuncture, acupressure [52], castor oil, bath or enema [53], and raspberry leaf [54]. As a physiological background, breast stimulation is considered to increase oxytocin levels, causing uterine contraction which then leads to the spontaneous onset of labor [55]. In fact, breast stimulation has also been shown to increase plasma oxytocin levels [56,57,58] and salivary oxytocin levels. The increase in the salivary oxytocin level after breast stimulation [55] is similar to the increase in the salivary oxytocin level after an aromatherapy footbath in the present study. Therefore, an aromatherapy footbath can be considered as having a stimulatory effect on the spontaneous onset of labor. 

Regarding breast stimulation, a previous study that investigated the effects of breast stimulation for three consecutive days and the changes in the salivary oxytocin level on the first and the third days showed that repeated breast stimulation increased the oxytocin level specifically on the third day. Considering our present findings and the results of previous studies, clary sage, lavender, and jasmine oils may exert gradual and accumulated effects on increasing the oxytocin level and stimulating the spontaneous onset of labor. In fact, repeated use of aromatherapy a few times a day or a week has been introduced to stimulate the spontaneous onset of labor [7,10,31]. Moreover, the repeated or continuous use of lavender or jasmine oil during labor until delivery shortened the duration of labor [9,15,16,17]. However, a short period of usage showed only a trend of shortening the duration of labor [59]. The duration of labor depends on uterine contraction, which is induced by oxytocin. Therefore, the repeated use of clary sage, lavender, and jasmine oils might gradually enhance the release of oxytocin. The effects of the repeated use of an aromatherapy footbath on the oxytocin level and the spontaneous onset of labor and the usage of an aromatherapy footbath in terms of frequency and duration per day or week warrant future investigation.

The footbath infused with clary sage and lavender oils and the footbath infused with jasmine oil induced an increase in the salivary oxytocin level. On the other hand, the footbath with no essential oils showed a decreasing trend in the salivary oxytocin level. However, the differences in the induced salivary oxytocin levels between the aromatherapy footbaths and the footbath with no essential oil were not significant. This lack of significant differences may have been caused by the large standard deviations of the changes in the salivary oxytocin levels. Notably, the standard deviations in the oxytocin level in the present study did not exceed those of previous studies [60,61]. However, the accumulation of deviations in the oxytocin level could result in large discrepancies in the changes in the oxytocin level. 

Previous studies have shown a wider range of oxytocin levels when measured without an extraction step than when measured with an extraction step [62,63]. In contrast, the latest ELISA kit has been improved, and lyophilization in the assay process can increase the validity of measuring the oxytocin level [47]. Our measurement of the oxytocin level by ELISA in the present study did not include an extraction step. To reduce oxytocin level deviation, an extraction step in the measurement of the oxytocin level is recommended.

### 4.2. Secondary Outcomes: Changes in Subjective Uterine Contraction and Salivary Cortisol Level

An increase in subjective uterine contractions in accordance with the increase in the salivary oxytocin level was not observed when using the footbath infused with clary sage and lavender oils. Our search for studies that investigated uterine contractions after the use of aromatherapy including clary sage, lavender, or jasmine oil in term pregnant women yielded no reports. Therefore, we could not compare our findings with other studies regarding the relations of uterine contractions, oxytocin, and aromatherapy. However, from a physiologic perspective, oxytocin causes uterine contractions by binding to oxytocin receptors. The concentration of oxytocin receptors was related to the responsiveness to the oxytocin change [64]. The concentration of oxytocin receptors was found to increase through pregnancy and reach a peak in early labor or 40 weeks of gestation with labor [12,65,66]. The mean gestation weeks of the women in the present study was 38, and thus they might not be responsive to the increase in the oxytocin level. To evaluate the effects of aromatherapy on uterine contractions and the spontaneous onset of labor, the responsiveness to the oxytocin increase of the participants needs to be considered. This means that in subsequent studies to evaluate uterine contractions in accordance with the changes in oxytocin levels, the participants should be enrolled from around 40 weeks of gestation.

Contrary to our supposition that the change in oxytocin levels of the participants using a footbath containing lavender oil will be larger than that using other footbaths, the salivary cortisol level significantly decreased after using each footbath regardless of whether lavender oil was infused or not. This indicates that a footbath alone can reduce stress, consistent with the finding of a previous study that examined the salivary cortisol level after spa bathing [67]. The use of lavender oil was expected to decrease the salivary cortisol level, and the changes in the salivary cortisol level were also likely associated with the changes in the salivary oxytocin level. There was no significant difference in the changes in the salivary cortisol levels between the footbath infused with clary sage and lavender oils and the footbath with no essential oil. The correlation between the changes in the salivary oxytocin and cortisol levels was very weak and not significant. Therefore, the increase in the salivary oxytocin level after using the footbath infused with clary sage and lavender oils could not likely be due to the suppression of stress using lavender oil. 

### 4.3. Implication for Clinical Practice

The increasing trend in the salivary oxytocin level without any adverse effects and the reduction in the salivary cortisol level show that the use of aromatherapy footbaths infused with clary sage and lavender oils and jasmine oil in the clinical setting can reduce stress in pregnant women.

### 4.4. Limitations and Strengths

This study has some limitations. First, the study design involved two interventions for each participant. Thus, the possibility of a carry-over effect to the outcomes of the second intervention from the first intervention cannot be completely excluded. Second, the estimated deviation in the changes in salivary oxytocin levels in the sample size calculation was very small. A large sample size is recommended in the comparison between groups regarding the change in the salivary oxytocin level. Third, we set a plain footbath as a control group. However, any scent that the participants recognized during the experimental intervention might also affect the outcomes. Fourth, we did not use randomization on the allocation of the participants. Although we orderly allocated the participants to the footbath groups, bias may not have been completely eliminated.

In this study, we focused on the changes in oxytocin levels to evaluate the effects of aromatherapy footbaths in term pregnant women as an index of stimulating the onset of labor. The results showed an increase in the oxytocin level after using the footbath with clary sage and lavender oils. Future studies need to evaluate the delivery outcomes.

In terms of strength and importance, this study is apparently the first to evaluate the physiology underlying the effects of aromatherapy footbaths prepared for enhancing the spontaneous onset of labor in term pregnant women using reproducible interventions. 

## 5. Conclusions

The salivary oxytocin level of term pregnant women significantly increased after using the footbath infused with clary sage and lavender oils. Moreover, the salivary oxytocin level showed an increasing trend after using the footbath infused with jasmine oil. These aromatherapy footbaths caused no adverse events regarding the pregnant women and their babies. In contrast, the salivary oxytocin level showed a decreasing trend after using the footbath with no essential oil. The changes in the pre-footbath and post-footbath salivary oxytocin levels in the experimental groups were not significantly different from the change in the pre-footbath and post-footbath salivary oxytocin level in the control group. Significant differences in changes in the subjective uterine contractions and salivary cortisol levels were not found between the experimental groups and the control group. The absence of a significant difference in salivary oxytocin levels from that of the control group may be due to the large deviation in changes in the salivary oxytocin level and the single use of an aromatherapy footbath. The repeated use of an aromatherapy footbath with improved measurement methods of the oxytocin level and the evaluation of delivery outcomes in a larger sample with randomized allocation are recommended.

## Figures and Tables

**Table 1:** Characteristics of participants according to intervention groups (GW, gestation weeks; USG, ultrasonography; BMI, body mass index; CES-D, Center for Epidemiologic Studies Depression Scale; STAI, State-Trait Anxiety Inventory).

	Clary Sage and Lavender Oil Group (*n* = 27)		Jasmine Oil Group (*n* = 26)		No Essential Oil Group (*n* = 25)		*p*-Value (vs. No Essential Oil Group)	
							Clary Sage and Lavender Oil Group	Jasmine Oil Group
Age (years) *	31.9	[4.1]	32.8	[4.0]	32.9	[2.8]	0.298	0.909
Gestation weeks *	38.6	[0.5]	38.8	[0.5]	38.6	[0.4]	0.894	0.163
Due date confirmed by USG ≤12 GW	25	(92.6)	25	(96.2)	25	(100)	0.491	1
Primiparas	19	(70.4)	12	(46.2)	16	(64.0)	0.625	0.2
Married	27	(100)	26	(100)	25	(100)	-	-
BMI before pregnancy ≥ 29	0	(0)	0	(0)	0	(0)	-	-
Country of origin: Japan	27	(100)	26	(100)	25	(100)	-	-
Education ≥ 12 years	27	(100)	24	(92.3)	23	(92.0)	0.226	1
Depression (CES-D score > 15)	1	(3.7)	1	(3.8)	0	(0)	0.519	0.51
Trait anxiety score (STAI) *	37.4	[7.3]	38.1	[6.8]	35.5	[5.3]	0.293	0.137
State anxiety score (STAI) *	32.5	[8.1]	34.3	[7.9]	32.4	[6.9]	0.97	0.363

*p*-value: using the two-tailed *t* test, χ^2^ test, or Fisher’s exact test of comparisons between clary sage and lavender oil group or jasmine oil group and no essential oil group; [ ] shows standard deviation; ( ) shows %. * Data are expressed as mean. Per-protocol analysis was conducted.

**Table 2:** Pre-footbath and post-footbath salivary oxytocin levels (pg/mL) according to intervention groups (SD, standard deviation; CI, confidence interval; MD, mean difference; df, degrees of freedom).

	Mean	SD	95% CI	MD	SD	95% CI	t	df	*p*-Value
Clary sage and lavender oil group (*n* = 19)									
Pre	139.5	106.5	[88.1, 190.8]	12.5	23.9	[1.0, 24.1]	2.29	18	0.035
Post	152.0	115.2	[96.5, 207.5]						
Jasmine oil group (*n* = 21)									
Pre	166.2	136.7	[104.0, 228.4]	4.8	51.8	[−18.8, 28.4]	0.43	20	0.676
Post	171.0	141.4	[106.6, 253.4]						
No essential oil group (*n* = 20)									
Pre	141.6	118.3	[86.3, 197.0]	−3.3	71.0	[−36.5, 29.9]	−0.21	19	0.839
Post	138.4	91.4	[95.6, 181.1]						

*p*-value: using the two-tailed paired *t*-test. Per-protocol analysis was conducted.

**Table 3:** Changes in pre-footbath and post-footbath subjective uterine contractions (%) according to intervention groups.

			*p*-Value
Clary sage and lavender oil group (*n* = 27)			
Increased	3	(11.1)	0.882
Unchanged	22	(81.5)	
Decreased	2	(7.4)	
Jasmine oil group (*n* = 26)			
Increased	7	(26.9)	0.544
Unchanged	17	(65.4)	
Decreased	2	(7.7)	
No essential oil group (*n* = 25)			
Increased	4	(16.0)	-
Unchanged	20	(80.0)	
Decreased	1	(4.0)	

*p*-value: using Fisher’s exact test of comparison between clary sage and lavender oil group or jasmine oil group versus no essential oil group. Per protocol analysis was conducted.

**Table 4:** Pre-footbath and post-footbath salivary cortisol levels (ng/mL) according to intervention groups (SD, standard deviation; CI, confidence interval; MD, mean difference; df, degrees of freedom).

	Mean	SD	95% CI	MD	SD	95% CI	t	df	*p*-Value
Clary sage and lavender oil group (*n* = 27)									
Pre	3.84	1.36	[3.30, 4.38]	−0.42	0.48	[−0.62, −0.24]	−4.61	26	<0.001
Post	3.41	1.15	[2.96, 3.87]						
Jasmine oil group (*n* = 26)									
Pre	3.80	1.01	[3.39, 4.21]	−0.41	0.53	[−0.63, −0.19]	−3.91	25	0.001
Post	3.39	0.80	[3.07, 3.71]						
No essential oil group (*n* = 24)									
Pre	3.90	1.14	[3.42, 4.38]	−0.40	0.39	[−0.56, −0.24]	−5.03	23	<0.001
Post	3.50	1.01	[3.07, 3.93]						

*p*-value: using the two-tailed paired *t*-test. Per protocol analysis was conducted.

## Data Availability

These data cannot be made publicly available since the participants did not provide their approval for the sharing of their data publicly. For researchers who meet the requirements for access to data, data are available from the corresponding author.
